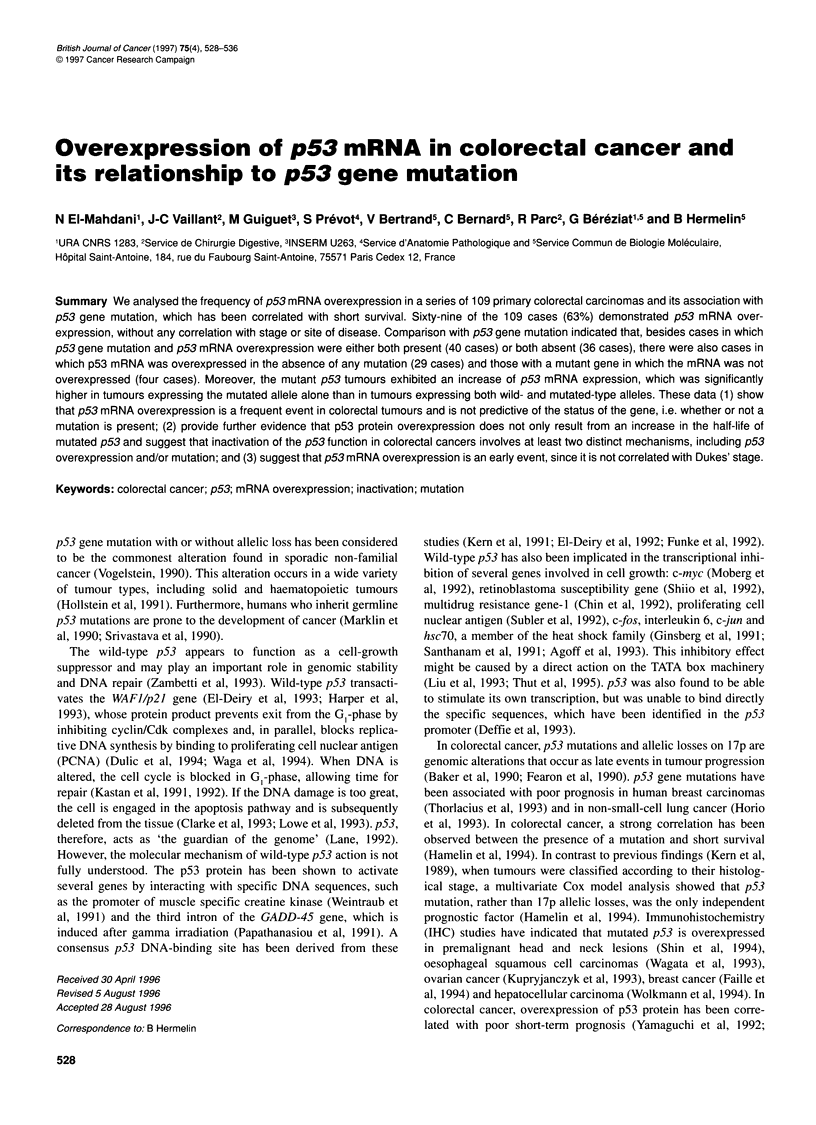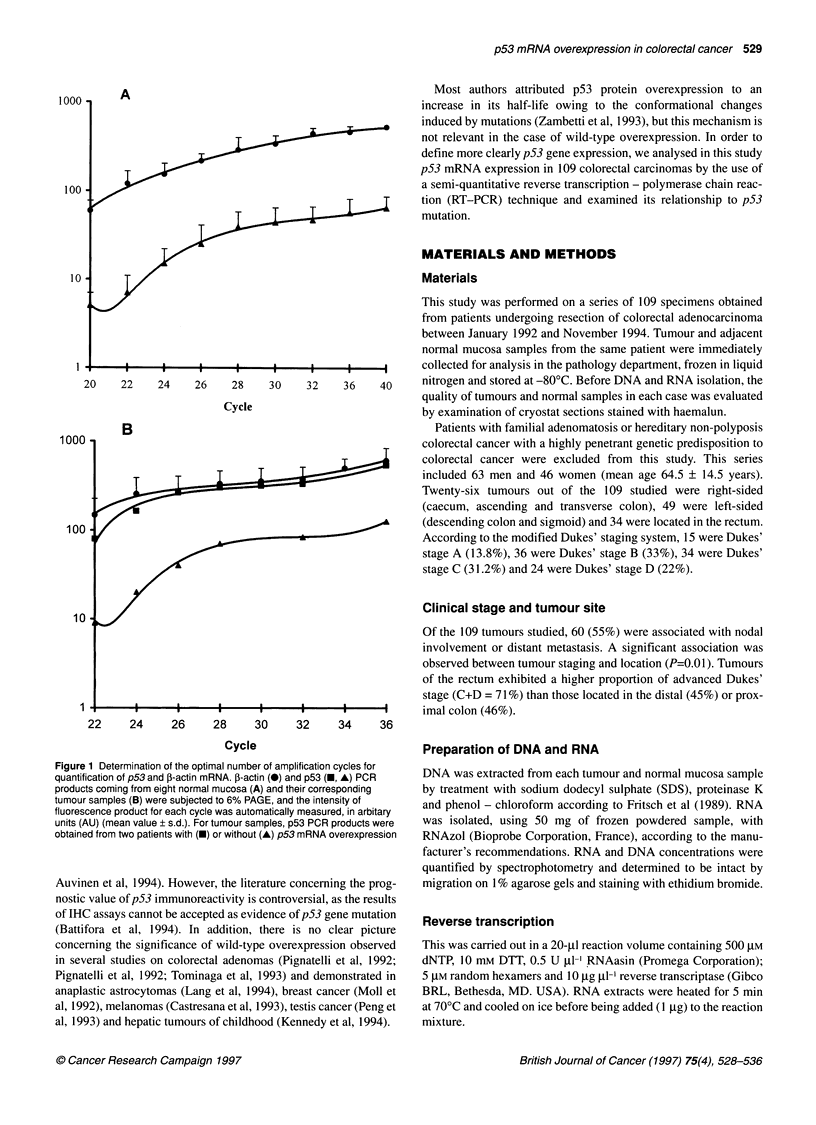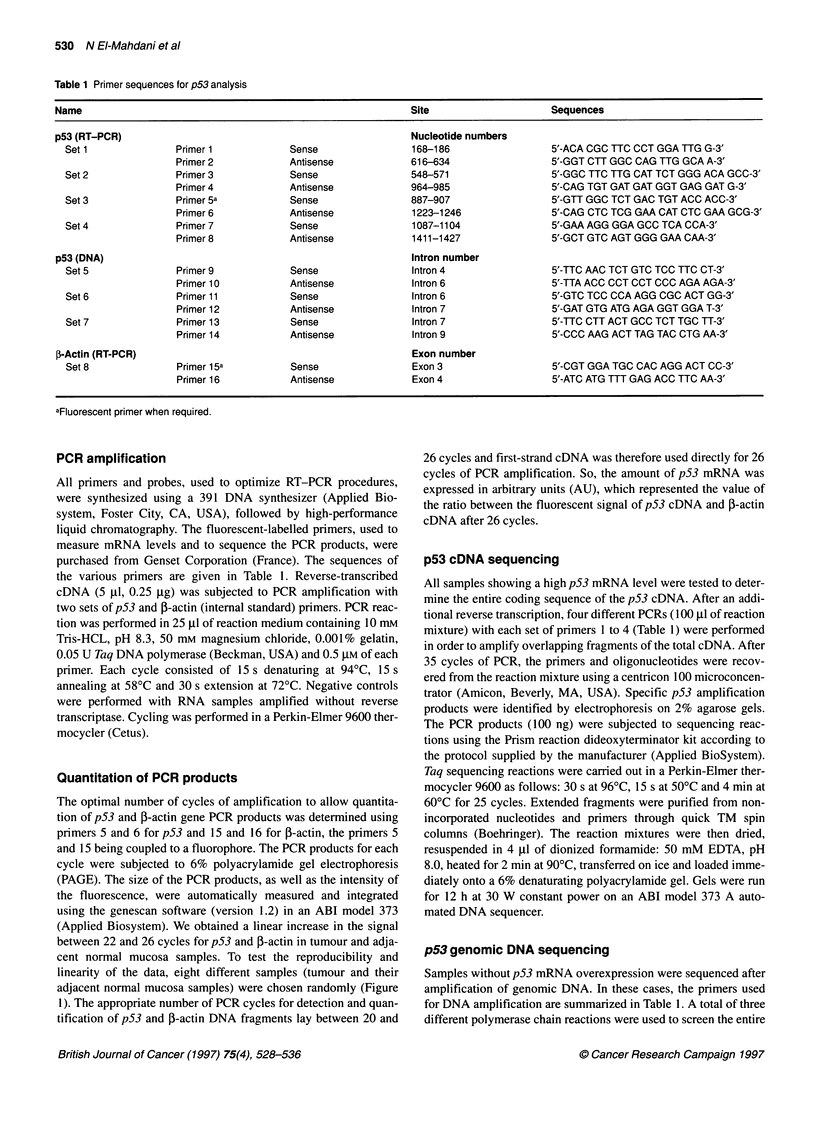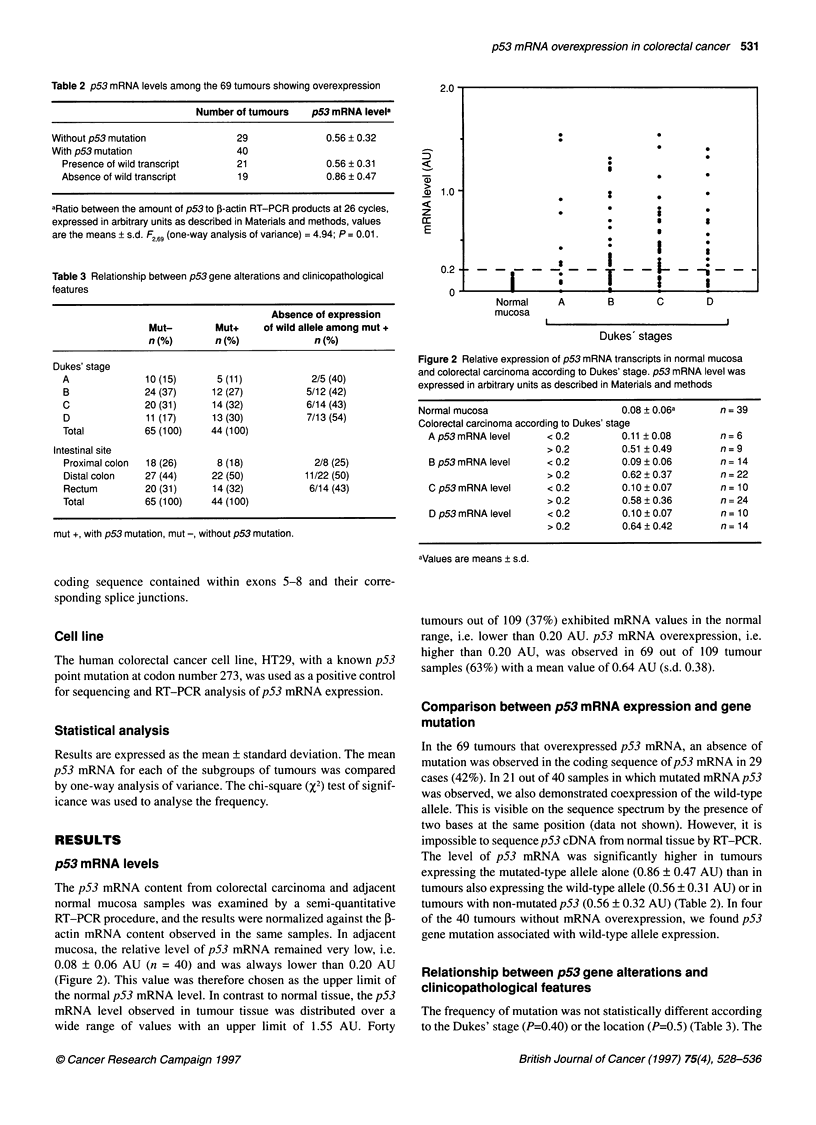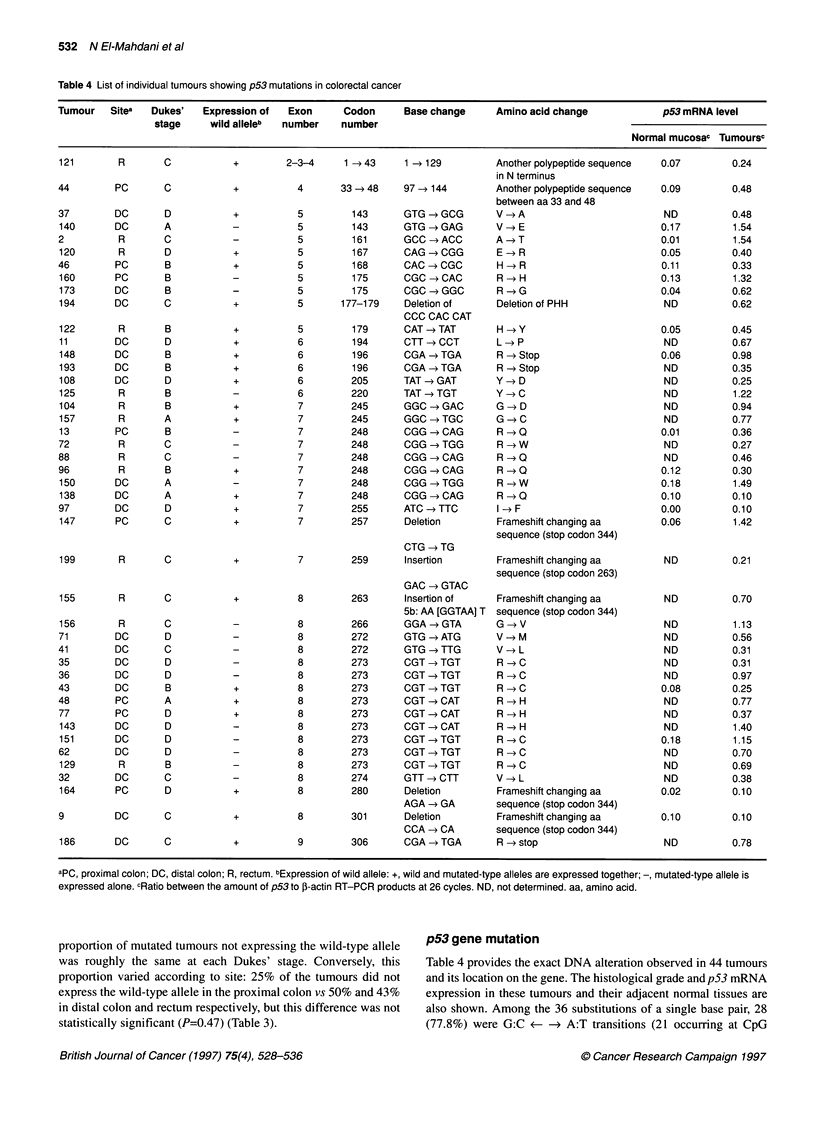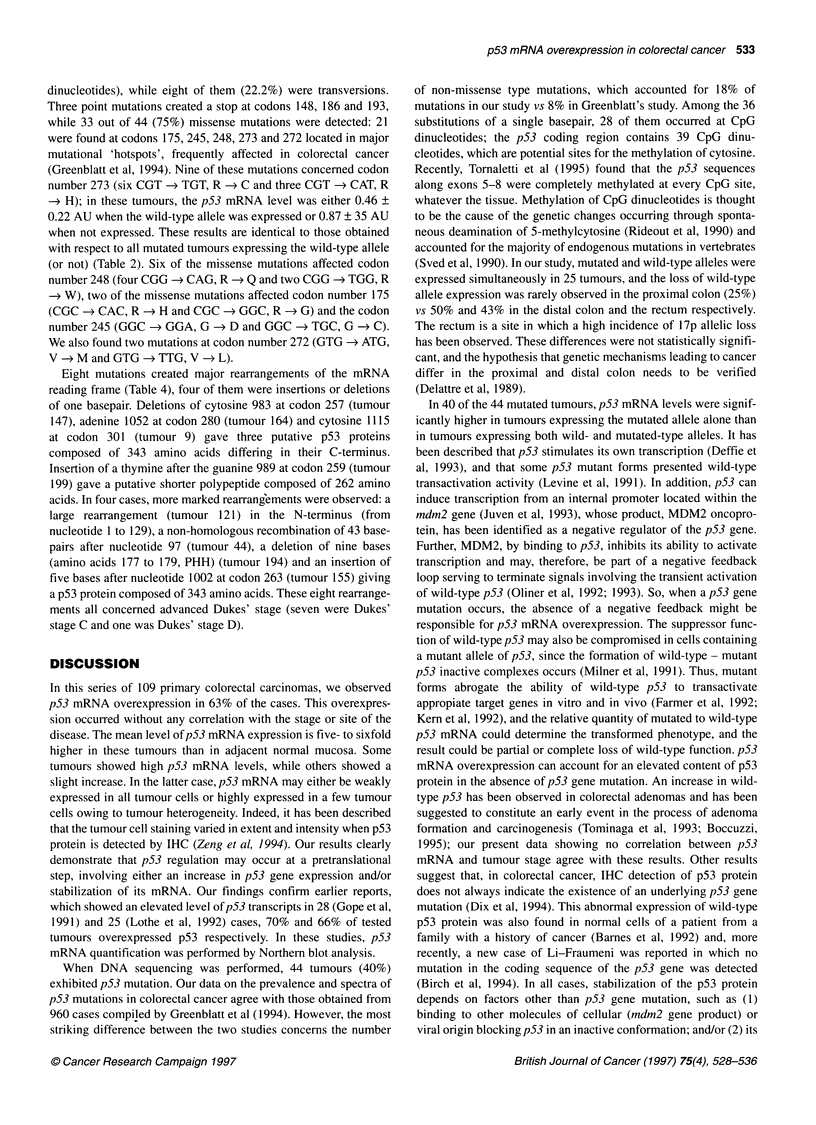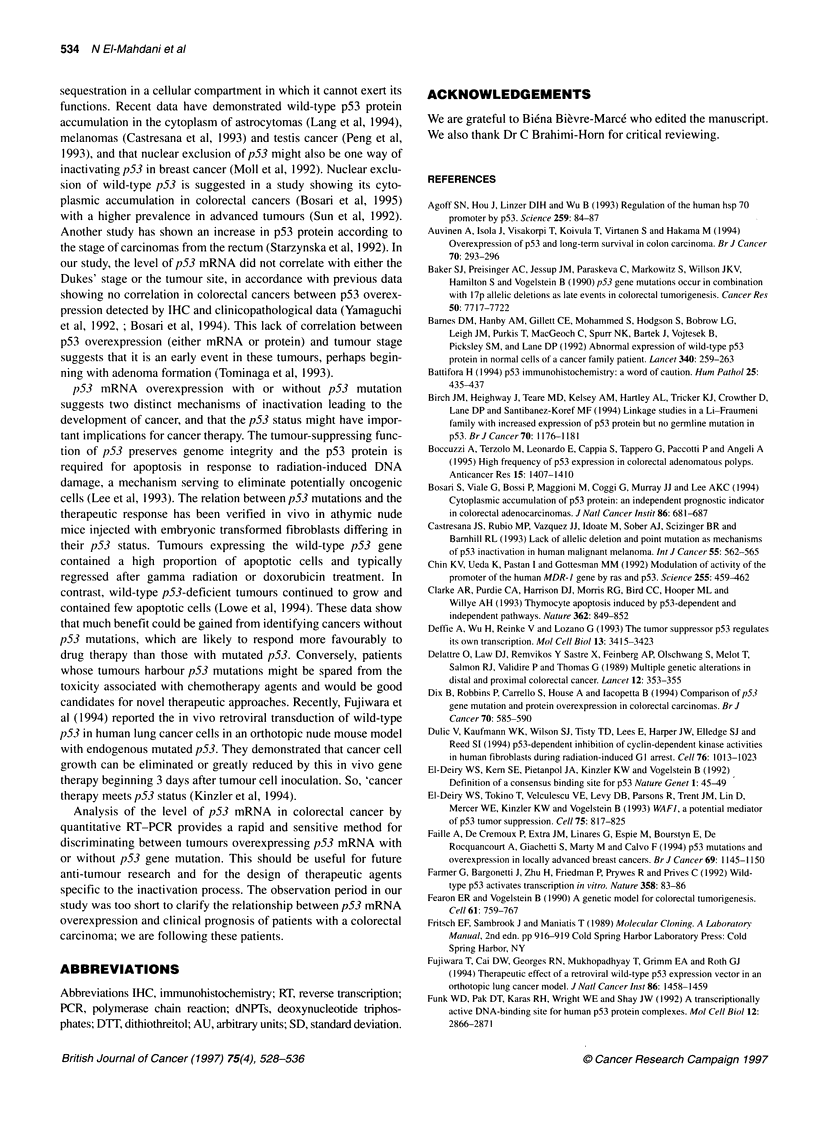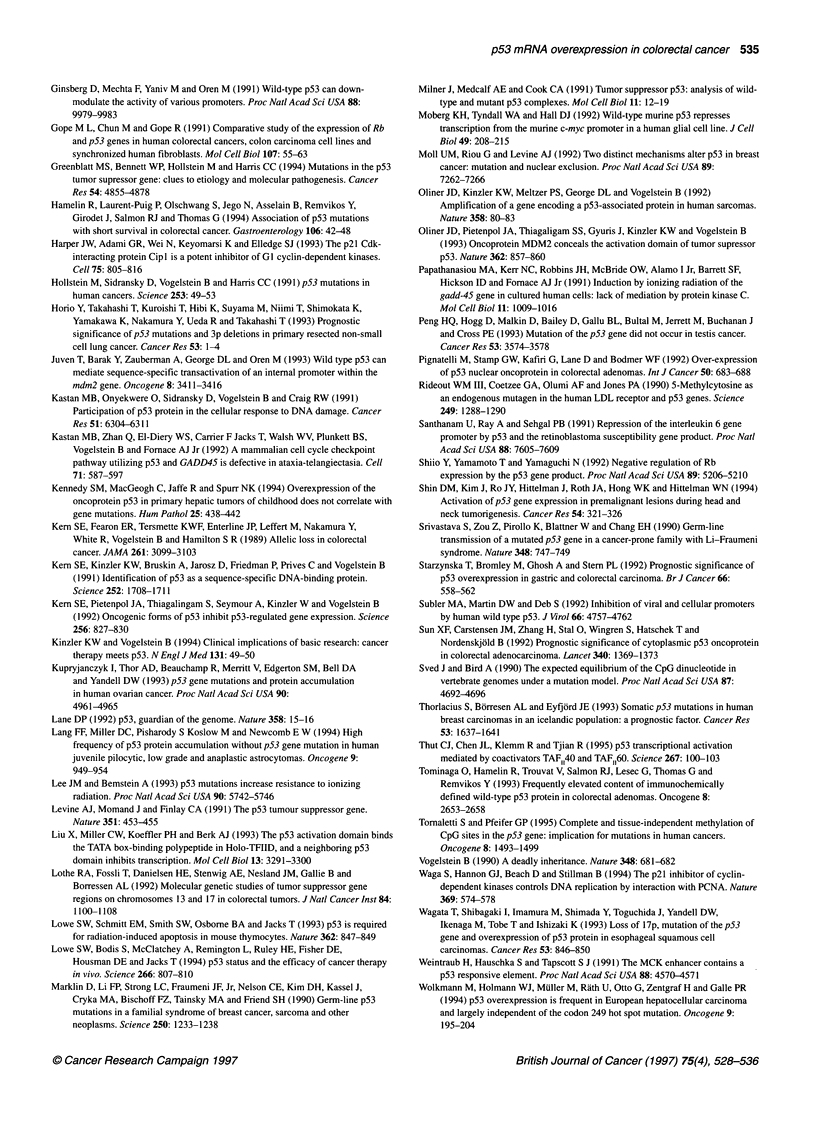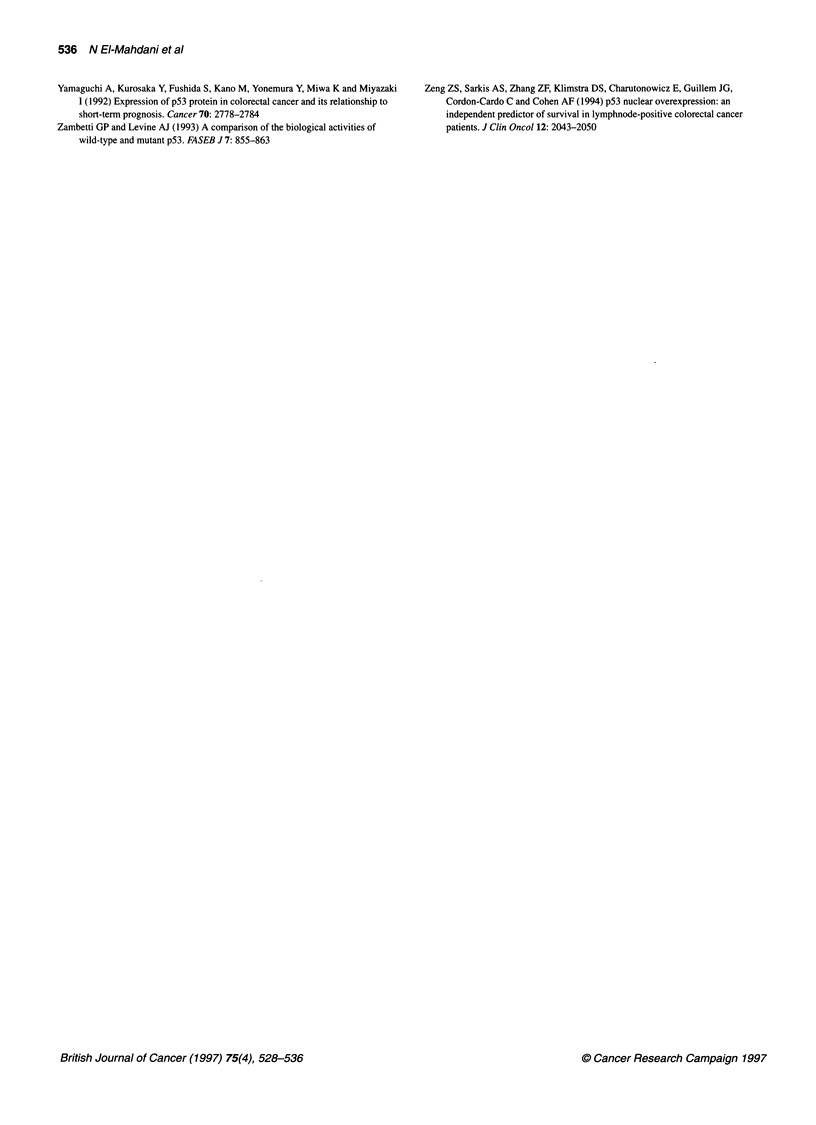# Overexpression of p53 mRNA in colorectal cancer and its relationship to p53 gene mutation.

**DOI:** 10.1038/bjc.1997.92

**Published:** 1997

**Authors:** N. el-Mahdani, J. C. Vaillant, M. Guiguet, S. Prévot, V. Bertrand, C. Bernard, R. Parc, G. Béréziat, B. Hermelin

**Affiliations:** URA CNRS 1283, Hôpital Saint-Antoine, Paris, France.

## Abstract

We analysed the frequency of p53 mRNA overexpression in a series of 109 primary colorectal carcinomas and its association with p53 gene mutation, which has been correlated with short survival. Sixty-nine of the 109 cases (63%) demonstrated p53 mRNA overexpression, without any correlation with stage or site of disease. Comparison with p53 gene mutation indicated that, besides cases in which p53 gene mutation and p53 mRNA overexpression were either both present (40 cases) or both absent (36 cases), there were also cases in which p53 mRNA was overexpressed in the absence of any mutation (29 cases) and those with a mutant gene in which the mRNA was not overexpressed (four cases). Moreover, the mutant p53 tumours exhibited an increase of p53 mRNA expression, which was significantly higher in tumours expressing the mutated allele alone than in tumours expressing both wild- and mutated-type alleles. These data (1) show that p53 mRNA overexpression is a frequent event in colorectal tumours and is not predictive of the status of the gene, i.e. whether or not a mutation is present; (2) provide further evidence that p53 protein overexpression does not only result from an increase in the half-life of mutated p53 and suggest that inactivation of the p53 function in colorectal cancers involves at least two distinct mechanisms, including p53 overexpression and/or mutation; and (3) suggest that p53 mRNA overexpression is an early event, since it is not correlated with Dukes stage.


British Journal of Cancer (1997) 75(4), 528–536
© 1997 Cancer Research Campaign

### Overexpression of *p53* mRNA in colorectal cancer and its relationship to *p53* gene mutation

**N El-Mahdani[1], J-C Vaillant[2], M Guiguet[3], S Prévot[4], V Bertrand[5], C Bernard[5], R Parc[2], G Béréziat[1,5] and B Hermelin[5]**

[1]URA CNRS 1283, [2]Service de Chirurgie Digestive, [3]INSERM U263, [4]Service d'Anatomie Pathologique and [5]Service Commun de Biologie Moléculaire, Hôpital Saint-Antoine, 184, rue du Faubourg Saint-Antoine, 75571 Paris Cedex 12, France

**Summary** We analysed the frequency of *p53* mRNA overexpression in a series of 109 primary colorectal carcinomas and its association with *p53* gene mutation, which has been correlated with short survival. Sixty-nine of the 109 cases (63%) demonstrated *p53* mRNA overexpression, without any correlation with stage or site of disease. Comparison with *p53* gene mutation indicated that, besides cases in which *p53* gene mutation and *p53* mRNA overexpression were either both present (40 cases) or both absent (36 cases), there were also cases in which p53 mRNA was overexpressed in the absence of any mutation (29 cases) and those with a mutant gene in which the mRNA was not overexpressed (four cases). Moreover, the mutant *p53* tumours exhibited an increase of *p53* mRNA expression, which was significantly higher in tumours expressing the mutated allele alone than in tumours expressing both wild- and mutated-type alleles. These data (1) show that *p53* mRNA overexpression is a frequent event in colorectal tumours and is not predictive of the status of the gene, i.e. whether or not a mutation is present; (2) provide further evidence that p53 protein overexpression does not only result from an increase in the half-life of mutated *p53* and suggest that inactivation of the *p53* function in colorectal cancers involves at least two distinct mechanisms, including *p53* overexpression and/or mutation; and (3) suggest that *p53* mRNA overexpression is an early event, since it is not correlated with Dukes' stage.

**Keywords:** colorectal cancer; *p53*; mRNA overexpression; inactivation; mutation

*p53* gene mutation with or without allelic loss has been considered to be the commonest alteration found in sporadic non-familial cancer (Vogelstein, 1990). This alteration occurs in a wide variety of tumour types, including solid and haematopoietic tumours (Hollstein et al, 1991). Furthermore, humans who inherit germline *p53* mutations are prone to the development of cancer (Marklin et al, 1990; Srivastava et al, 1990).

The wild-type *p53* appears to function as a cell-growth suppressor and may play an important role in genomic stability and DNA repair (Zambetti et al, 1993). Wild-type *p53* transactivates the *WAF1/p21* gene (El-Deiry et al, 1993; Harper et al, 1993), whose protein product prevents exit from the $G_1$-phase by inhibiting cyclin/Cdk complexes and, in parallel, blocks replicative DNA synthesis by binding to proliferating cell nuclear antigen (PCNA) (Dulic et al, 1994; Waga et al, 1994). When DNA is altered, the cell cycle is blocked in $G_1$-phase, allowing time for repair (Kastan et al, 1991, 1992). If the DNA damage is too great, the cell is engaged in the apoptosis pathway and is subsequently deleted from the tissue (Clarke et al, 1993; Lowe et al, 1993). *p53*, therefore, acts as 'the guardian of the genome' (Lane, 1992). However, the molecular mechanism of wild-type *p53* action is not fully understood. The p53 protein has been shown to activate several genes by interacting with specific DNA sequences, such as the promoter of muscle specific creatine kinase (Weintraub et al, 1991) and the third intron of the *GADD-45* gene, which is induced after gamma irradiation (Papathanasiou et al, 1991). A consensus *p53* DNA-binding site has been derived from these studies (Kern et al, 1991; El-Deiry et al, 1992; Funke et al, 1992). Wild-type *p53* has also been implicated in the transcriptional inhibition of several genes involved in cell growth: c-*myc* (Moberg et al, 1992), retinoblastoma susceptibility gene (Shiio et al, 1992), multidrug resistance gene-1 (Chin et al, 1992), proliferating cell nuclear antigen (Subler et al, 1992), c-*fos*, interleukin 6, c-*jun* and *hsc*70, a member of the heat shock family (Ginsberg et al, 1991; Santhanam et al, 1991; Agoff et al, 1993). This inhibitory effect might be caused by a direct action on the TATA box machinery (Liu et al, 1993; Thut et al, 1995). *p53* was also found to be able to stimulate its own transcription, but was unable to bind directly the specific sequences, which have been identified in the *p53* promoter (Deffie et al, 1993).

*Received 30 April 1996*
*Revised 5 August 1996*
*Accepted 28 August 1996*

*Correspondence to:* B Hermelin

In colorectal cancer, *p53* mutations and allelic losses on 17p are genomic alterations that occur as late events in tumour progression (Baker et al, 1990; Fearon et al, 1990). *p53* gene mutations have been associated with poor prognosis in human breast carcinomas (Thorlacius et al, 1993) and in non-small-cell lung cancer (Horio et al, 1993). In colorectal cancer, a strong correlation has been observed between the presence of a mutation and short survival (Hamelin et al, 1994). In contrast to previous findings (Kern et al, 1989), when tumours were classified according to their histological stage, a multivariate Cox model analysis showed that *p53* mutation, rather than 17p allelic losses, was the only independent prognostic factor (Hamelin et al, 1994). Immunohistochemistry (IHC) studies have indicated that mutated *p53* is overexpressed in premalignant head and neck lesions (Shin et al, 1994), oesophageal squamous cell carcinomas (Wagata et al, 1993), ovarian cancer (Kupryjanczyk et al, 1993), breast cancer (Faille et al, 1994) and hepatocellular carcinoma (Wolkmann et al, 1994). In colorectal cancer, overexpression of p53 protein has been correlated with poor short-term prognosis (Yamaguchi et al, 1992;

**Figure 1** Determination of the optimal number of amplification cycles for quantification of *p53* and β-actin mRNA. β-actin (●) and p53 (■, ▲) PCR products coming from eight normal mucosa (**A**) and their corresponding tumour samples (**B**) were subjected to 6% PAGE, and the intensity of fluorescence product for each cycle was automatically measured, in arbitary units (AU) (mean value ± s.d.). For tumour samples, p53 PCR products were obtained from two patients with (■) or without (▲) *p53* mRNA overexpression

Auvinen et al, 1994). However, the literature concerning the prognostic value of *p53* immunoreactivity is controversial, as the results of IHC assays cannot be accepted as evidence of *p53* gene mutation (Battifora et al, 1994). In addition, there is no clear picture concerning the significance of wild-type overexpression observed in several studies on colorectal adenomas (Pignatelli et al, 1992; Pignatelli et al, 1992; Tominaga et al, 1993) and demonstrated in anaplastic astrocytomas (Lang et al, 1994), breast cancer (Moll et al, 1992), melanomas (Castresana et al, 1993), testis cancer (Peng et al, 1993) and hepatic tumours of childhood (Kennedy et al, 1994).

Most authors attributed p53 protein overexpression to an increase in its half-life owing to the conformational changes induced by mutations (Zambetti et al, 1993), but this mechanism is not relevant in the case of wild-type overexpression. In order to define more clearly *p53* gene expression, we analysed in this study *p53* mRNA expression in 109 colorectal carcinomas by the use of a semi-quantitative reverse transcription – polymerase chain reaction (RT–PCR) technique and examined its relationship to *p53* mutation.

### MATERIALS AND METHODS

### Materials

This study was performed on a series of 109 specimens obtained from patients undergoing resection of colorectal adenocarcinoma between January 1992 and November 1994. Tumour and adjacent normal mucosa samples from the same patient were immediately collected for analysis in the pathology department, frozen in liquid nitrogen and stored at –80°C. Before DNA and RNA isolation, the quality of tumours and normal samples in each case was evaluated by examination of cryostat sections stained with haemalun.

Patients with familial adenomatosis or hereditary non-polyposis colorectal cancer with a highly penetrant genetic predisposition to colorectal cancer were excluded from this study. This series included 63 men and 46 women (mean age 64.5 ± 14.5 years). Twenty-six tumours out of the 109 studied were right-sided (caecum, ascending and transverse colon), 49 were left-sided (descending colon and sigmoid) and 34 were located in the rectum. According to the modified Dukes' staging system, 15 were Dukes' stage A (13.8%), 36 were Dukes' stage B (33%), 34 were Dukes' stage C (31.2%) and 24 were Dukes' stage D (22%).

### Clinical stage and tumour site

Of the 109 tumours studied, 60 (55%) were associated with nodal involvement or distant metastasis. A significant association was observed between tumour staging and location ($P$=0.01). Tumours of the rectum exhibited a higher proportion of advanced Dukes' stage (C+D = 71%) than those located in the distal (45%) or proximal colon (46%).

### Preparation of DNA and RNA

DNA was extracted from each tumour and normal mucosa sample by treatment with sodium dodecyl sulphate (SDS), proteinase K and phenol – chloroform according to Fritsch et al (1989). RNA was isolated, using 50 mg of frozen powdered sample, with RNAzol (Bioprobe Corporation, France), according to the manufacturer's recommendations. RNA and DNA concentrations were quantified by spectrophotometry and determined to be intact by migration on 1% agarose gels and staining with ethidium bromide.

### Reverse transcription

This was carried out in a 20-μl reaction volume containing 500 μM dNTP, 10 mM DTT, 0.5 U $\mu l^{-1}$ RNAasin (Promega Corporation); 5 μM random hexamers and 10 μg $\mu l^{-1}$ reverse transcriptase (Gibco BRL, Bethesda, MD. USA). RNA extracts were heated for 5 min at 70°C and cooled on ice before being added (1 μg) to the reaction mixture.

**Table 1** Primer sequences for *p53* analysis

| Name | | | Site | Sequences |
|---|---|---|---|---|
| **p53 (RT–PCR)** | | | **Nucleotide numbers** | |
| Set 1 | Primer 1 | Sense | 168–186 | 5′-ACA CGC TTC CCT GGA TTG G-3′ |
| | Primer 2 | Antisense | 616–634 | 5′-GGT CTT GGC CAG TTG GCA A-3′ |
| Set 2 | Primer 3 | Sense | 548–571 | 5′-GGC TTC TTG CAT TCT GGG ACA GCC-3′ |
| | Primer 4 | Antisense | 964–985 | 5′-CAG TGT GAT GAT GGT GAG GAT G-3′ |
| Set 3 | Primer 5[a] | Sense | 887–907 | 5′-GTT GGC TCT GAC TGT ACC ACC-3′ |
| | Primer 6 | Antisense | 1223–1246 | 5′-CAG CTC TCG GAA CAT CTC GAA GCG-3′ |
| Set 4 | Primer 7 | Sense | 1087–1104 | 5′-GAA AGG GGA GCC TCA CCA-3′ |
| | Primer 8 | Antisense | 1411–1427 | 5′-GCT GTC AGT GGG GAA CAA-3′ |
| **p53 (DNA)** | | | **Intron number** | |
| Set 5 | Primer 9 | Sense | Intron 4 | 5′-TTC AAC TCT GTC TCC TTC CT-3′ |
| | Primer 10 | Antisense | Intron 6 | 5′-TTA ACC CCT CCT CCC AGA AGA-3′ |
| Set 6 | Primer 11 | Sense | Intron 6 | 5′-GTC TCC CCA AGG CGC ACT GG-3′ |
| | Primer 12 | Antisense | Intron 7 | 5′-GAT GTG ATG AGA GGT GGA T-3′ |
| Set 7 | Primer 13 | Sense | Intron 7 | 5′-TTC CTT ACT GCC TCT TGC TT-3′ |
| | Primer 14 | Antisense | Intron 9 | 5′-CCC AAG ACT TAG TAC CTG AA-3′ |
| **β-Actin (RT-PCR)** | | | **Exon number** | |
| Set 8 | Primer 15[a] | Sense | Exon 3 | 5′-CGT GGA TGC CAC AGG ACT CC-3′ |
| | Primer 16 | Antisense | Exon 4 | 5′-ATC ATG TTT GAG ACC TTC AA-3′ |

[a]Fluorescent primer when required.

### PCR amplification

All primers and probes, used to optimize RT–PCR procedures, were synthesized using a 391 DNA synthesizer (Applied Biosystem, Foster City, CA, USA), followed by high-performance liquid chromatography. The fluorescent-labelled primers, used to measure mRNA levels and to sequence the PCR products, were purchased from Genset Corporation (France). The sequences of the various primers are given in Table 1. Reverse-transcribed cDNA (5 μl, 0.25 μg) was subjected to PCR amplification with two sets of *p53* and β-actin (internal standard) primers. PCR reaction was performed in 25 μl of reaction medium containing 10 mM Tris-HCL, pH 8.3, 50 mM magnesium chloride, 0.001% gelatin, 0.05 U *Taq* DNA polymerase (Beckman, USA) and 0.5 μM of each primer. Each cycle consisted of 15 s denaturing at 94°C, 15 s annealing at 58°C and 30 s extension at 72°C. Negative controls were performed with RNA samples amplified without reverse transcriptase. Cycling was performed in a Perkin-Elmer 9600 thermocycler (Cetus).

### Quantitation of PCR products

The optimal number of cycles of amplification to allow quantitation of *p53* and β-actin gene PCR products was determined using primers 5 and 6 for *p53* and 15 and 16 for β-actin, the primers 5 and 15 being coupled to a fluorophore. The PCR products for each cycle were subjected to 6% polyacrylamide gel electrophoresis (PAGE). The size of the PCR products, as well as the intensity of the fluorescence, were automatically measured and integrated using the genescan software (version 1.2) in an ABI model 373 (Applied Biosystem). We obtained a linear increase in the signal between 22 and 26 cycles for *p53* and β-actin in tumour and adjacent normal mucosa samples. To test the reproducibility and linearity of the data, eight different samples (tumour and their adjacent normal mucosa samples) were chosen randomly (Figure 1). The appropriate number of PCR cycles for detection and quantification of *p53* and β-actin DNA fragments lay between 20 and 26 cycles and first-strand cDNA was therefore used directly for 26 cycles of PCR amplification. So, the amount of *p53* mRNA was expressed in arbitrary units (AU), which represented the value of the ratio between the fluorescent signal of *p53* cDNA and β-actin cDNA after 26 cycles.

### p53 cDNA sequencing

All samples showing a high *p53* mRNA level were tested to determine the entire coding sequence of the *p53* cDNA. After an additional reverse transcription, four different PCRs (100 μl of reaction mixture) with each set of primers 1 to 4 (Table 1) were performed in order to amplify overlapping fragments of the total cDNA. After 35 cycles of PCR, the primers and oligonucleotides were recovered from the reaction mixture using a centricon 100 microconcentrator (Amicon, Beverly, MA, USA). Specific *p53* amplification products were identified by electrophoresis on 2% agarose gels. The PCR products (100 ng) were subjected to sequencing reactions using the Prism reaction dideoxyterminator kit according to the protocol supplied by the manufacturer (Applied BioSystem). *Taq* sequencing reactions were carried out in a Perkin-Elmer thermocycler 9600 as follows: 30 s at 96°C, 15 s at 50°C and 4 min at 60°C for 25 cycles. Extended fragments were purified from non-incorporated nucleotides and primers through quick TM spin columns (Boehringer). The reaction mixtures were then dried, resuspended in 4 μl of dionized formamide: 50 mM EDTA, pH 8.0, heated for 2 min at 90°C, transferred on ice and loaded immediately onto a 6% denaturating polyacrylamide gel. Gels were run for 12 h at 30 W constant power on an ABI model 373 A automated DNA sequencer.

### *p53* genomic DNA sequencing

Samples without *p53* mRNA overexpression were sequenced after amplification of genomic DNA. In these cases, the primers used for DNA amplification are summarized in Table 1. A total of three different polymerase chain reactions were used to screen the entire

 © Cancer Research Campaign 1997

**Table 2** *p53* mRNA levels among the 69 tumours showing overexpression

| | Number of tumours | *p53* mRNA level[a] |
|---|---|---|
| Without *p53* mutation | 29 | 0.56 ± 0.32 |
| With *p53* mutation | 40 | |
| Presence of wild transcript | 21 | 0.56 ± 0.31 |
| Absence of wild transcript | 19 | 0.86 ± 0.47 |

[a]Ratio between the amount of *p53* to β-actin RT–PCR products at 26 cycles, expressed in arbitrary units as described in Materials and methods, values are the means ± s.d. $F_{2,69}$ (one-way analysis of variance) = 4.94; $P = 0.01$.

**Table 3** Relationship between *p53* gene alterations and clinicopathological features

| | Mut– n (%) | Mut+ n (%) | Absence of expression of wild allele among mut + n (%) |
|---|---|---|---|
| Dukes' stage | | | |
| A | 10 (15) | 5 (11) | 2/5 (40) |
| B | 24 (37) | 12 (27) | 5/12 (42) |
| C | 20 (31) | 14 (32) | 6/14 (43) |
| D | 11 (17) | 13 (30) | 7/13 (54) |
| Total | 65 (100) | 44 (100) | |
| Intestinal site | | | |
| Proximal colon | 18 (26) | 8 (18) | 2/8 (25) |
| Distal colon | 27 (44) | 22 (50) | 11/22 (50) |
| Rectum | 20 (31) | 14 (32) | 6/14 (43) |
| Total | 65 (100) | 44 (100) | |

mut +, with *p53* mutation, mut –, without *p53* mutation.

**Figure 2** Relative expression of *p53* mRNA transcripts in normal mucosa and colorectal carcinoma according to Dukes' stage. *p53* mRNA level was expressed in arbitrary units as described in Materials and methods

| | | | |
|---|---|---|---|
| Normal mucosa | | 0.08 ± 0.06[a] | n = 39 |
| Colorectal carcinoma according to Dukes' stage | | | |
| A *p53* mRNA level | < 0.2 | 0.11 ± 0.08 | n = 6 |
| | > 0.2 | 0.51 ± 0.49 | n = 9 |
| B *p53* mRNA level | < 0.2 | 0.09 ± 0.06 | n = 14 |
| | > 0.2 | 0.62 ± 0.37 | n = 22 |
| C *p53* mRNA level | < 0.2 | 0.10 ± 0.07 | n = 10 |
| | > 0.2 | 0.58 ± 0.36 | n = 24 |
| D *p53* mRNA level | < 0.2 | 0.10 ± 0.07 | n = 10 |
| | > 0.2 | 0.64 ± 0.42 | n = 14 |

[a]Values are means ± s.d.

coding sequence contained within exons 5–8 and their corresponding splice junctions.

### Cell line

The human colorectal cancer cell line, HT29, with a known *p53* point mutation at codon number 273, was used as a positive control for sequencing and RT–PCR analysis of *p53* mRNA expression.

### Statistical analysis

Results are expressed as the mean ± standard deviation. The mean *p53* mRNA for each of the subgroups of tumours was compared by one-way analysis of variance. The chi-square ($\chi^2$) test of significance was used to analyse the frequency.

### RESULTS

### *p53* mRNA levels

The *p53* mRNA content from colorectal carcinoma and adjacent normal mucosa samples was examined by a semi-quantitative RT–PCR procedure, and the results were normalized against the β-actin mRNA content observed in the same samples. In adjacent mucosa, the relative level of *p53* mRNA remained very low, i.e. 0.08 ± 0.06 AU ($n$ = 40) and was always lower than 0.20 AU (Figure 2). This value was therefore chosen as the upper limit of the normal *p53* mRNA level. In contrast to normal tissue, the *p53* mRNA level observed in tumour tissue was distributed over a wide range of values with an upper limit of 1.55 AU. Forty tumours out of 109 (37%) exhibited mRNA values in the normal range, i.e. lower than 0.20 AU. *p53* mRNA overexpression, i.e. higher than 0.20 AU, was observed in 69 out of 109 tumour samples (63%) with a mean value of 0.64 AU (s.d. 0.38).

### Comparison between *p53* mRNA expression and gene mutation

In the 69 tumours that overexpressed *p53* mRNA, an absence of mutation was observed in the coding sequence of *p53* mRNA in 29 cases (42%). In 21 out of 40 samples in which mutated mRNA *p53* was observed, we also demonstrated coexpression of the wild-type allele. This is visible on the sequence spectrum by the presence of two bases at the same position (data not shown). However, it is impossible to sequence *p53* cDNA from normal tissue by RT–PCR. The level of *p53* mRNA was significantly higher in tumours expressing the mutated-type allele alone (0.86 ± 0.47 AU) than in tumours also expressing the wild-type allele (0.56 ± 0.31 AU) or in tumours with non-mutated *p53* (0.56 ± 0.32 AU) (Table 2). In four of the 40 tumours without mRNA overexpression, we found *p53* gene mutation associated with wild-type allele expression.

### Relationship between *p53* gene alterations and clinicopathological features

The frequency of mutation was not statistically different according to the Dukes' stage ($P$=0.40) or the location ($P$=0.5) (Table 3). The

© Cancer Research Campaign 1997

**Table 4** List of individual tumours showing *p53* mutations in colorectal cancer

| Tumour | Site[a] | Dukes' stage | Expression of wild allele[b] | Exon number | Codon number | Base change | Amino acid change | *p53* mRNA level | |
|---|---|---|---|---|---|---|---|---|---|
| | | | | | | | | Normal mucosa[c] | Tumours[c] |
| 121 | R | C | + | 2–3–4 | 1 → 43 | 1 → 129 | Another polypeptide sequence in N terminus | 0.07 | 0.24 |
| 44 | PC | C | + | 4 | 33 → 48 | 97 → 144 | Another polypeptide sequence between aa 33 and 48 | 0.09 | 0.48 |
| 37 | DC | D | + | 5 | 143 | GTG → GCG | V → A | ND | 0.48 |
| 140 | DC | A | – | 5 | 143 | GTG → GAG | V → E | 0.17 | 1.54 |
| 2 | R | C | – | 5 | 161 | GCC → ACC | A → T | 0.01 | 1.54 |
| 120 | R | D | + | 5 | 167 | CAG → CGG | E → R | 0.05 | 0.40 |
| 46 | PC | B | + | 5 | 168 | CAC → CGC | H → R | 0.11 | 0.33 |
| 160 | PC | B | – | 5 | 175 | CGC → CAC | R → H | 0.13 | 1.32 |
| 173 | DC | B | – | 5 | 175 | CGC → GGC | R → G | 0.04 | 0.62 |
| 194 | DC | C | + | 5 | 177–179 | Deletion of CCC CAC CAT | Deletion of PHH | ND | 0.62 |
| 122 | R | B | + | 5 | 179 | CAT → TAT | H → Y | 0.05 | 0.45 |
| 11 | DC | D | + | 6 | 194 | CTT → CCT | L → P | ND | 0.67 |
| 148 | DC | B | + | 6 | 196 | CGA → TGA | R → Stop | 0.06 | 0.98 |
| 193 | DC | B | + | 6 | 196 | CGA → TGA | R → Stop | ND | 0.35 |
| 108 | DC | D | + | 6 | 205 | TAT → GAT | Y → D | ND | 0.25 |
| 125 | R | B | – | 6 | 220 | TAT → TGT | Y → C | ND | 1.22 |
| 104 | R | B | + | 7 | 245 | GGC → GAC | G → D | ND | 0.94 |
| 157 | R | A | + | 7 | 245 | GGC → TGC | G → C | ND | 0.77 |
| 13 | PC | B | – | 7 | 248 | CGG → CAG | R → Q | 0.01 | 0.36 |
| 72 | R | C | – | 7 | 248 | CGG → TGG | R → W | ND | 0.27 |
| 88 | R | C | – | 7 | 248 | CGG → CAG | R → Q | ND | 0.46 |
| 96 | R | B | + | 7 | 248 | CGG → CAG | R → Q | 0.12 | 0.30 |
| 150 | DC | A | – | 7 | 248 | CGG → TGG | R → W | 0.18 | 1.49 |
| 138 | DC | A | + | 7 | 248 | CGG → CAG | R → Q | 0.10 | 0.10 |
| 97 | DC | D | + | 7 | 255 | ATC → TTC | I → F | 0.00 | 0.10 |
| 147 | PC | C | + | 7 | 257 | Deletion CTG → TG | Frameshift changing aa sequence (stop codon 344) | 0.06 | 1.42 |
| 199 | R | C | + | 7 | 259 | Insertion GAC → GTAC | Frameshift changing aa sequence (stop codon 263) | ND | 0.21 |
| 155 | R | C | + | 8 | 263 | Insertion of 5b: AA [GGTAA] T | Frameshift changing aa sequence (stop codon 344) | ND | 0.70 |
| 156 | R | C | – | 8 | 266 | GGA → GTA | G → V | ND | 1.13 |
| 71 | DC | D | – | 8 | 272 | GTG → ATG | V → M | ND | 0.56 |
| 41 | DC | C | – | 8 | 272 | GTG → TTG | V → L | ND | 0.31 |
| 35 | DC | D | – | 8 | 273 | CGT → TGT | R → C | ND | 0.31 |
| 36 | DC | D | – | 8 | 273 | CGT → TGT | R → C | ND | 0.97 |
| 43 | DC | B | + | 8 | 273 | CGT → TGT | R → C | 0.08 | 0.25 |
| 48 | PC | A | + | 8 | 273 | CGT → CAT | R → H | ND | 0.77 |
| 77 | PC | D | + | 8 | 273 | CGT → CAT | R → H | ND | 0.37 |
| 143 | DC | D | – | 8 | 273 | CGT → CAT | R → H | ND | 1.40 |
| 151 | DC | D | – | 8 | 273 | CGT → TGT | R → C | 0.18 | 1.15 |
| 62 | DC | D | – | 8 | 273 | CGT → TGT | R → C | ND | 0.70 |
| 129 | R | B | – | 8 | 273 | CGT → TGT | R → C | ND | 0.69 |
| 32 | DC | C | – | 8 | 274 | GTT → CTT | V → L | ND | 0.38 |
| 164 | PC | D | + | 8 | 280 | Deletion AGA → GA | Frameshift changing aa sequence (stop codon 344) | 0.02 | 0.10 |
| 9 | DC | C | + | 8 | 301 | Deletion CCA → CA | Frameshift changing aa sequence (stop codon 344) | 0.10 | 0.10 |
| 186 | DC | C | + | 9 | 306 | CGA → TGA | R → stop | ND | 0.78 |

[a]PC, proximal colon; DC, distal colon; R, rectum. [b]Expression of wild allele: +, wild and mutated-type alleles are expressed together; –, mutated-type allele is expressed alone. [c]Ratio between the amount of *p53* to β-actin RT–PCR products at 26 cycles. ND, not determined. aa, amino acid.

proportion of mutated tumours not expressing the wild-type allele was roughly the same at each Dukes' stage. Conversely, this proportion varied according to site: 25% of the tumours did not express the wild-type allele in the proximal colon *vs* 50% and 43% in distal colon and rectum respectively, but this difference was not statistically significant (*P*=0.47) (Table 3).

### *p53* gene mutation

Table 4 provides the exact DNA alteration observed in 44 tumours and its location on the gene. The histological grade and *p53* mRNA expression in these tumours and their adjacent normal tissues are also shown. Among the 36 substitutions of a single base pair, 28 (77.8%) were G:C ← → A:T transitions (21 occurring at CpG

 © Cancer Research Campaign 1997

dinucleotides), while eight of them (22.2%) were transversions. Three point mutations created a stop at codons 148, 186 and 193, while 33 out of 44 (75%) missense mutations were detected: 21 were found at codons 175, 245, 248, 273 and 272 located in major mutational 'hotspots', frequently affected in colorectal cancer (Greenblatt et al, 1994). Nine of these mutations concerned codon number 273 (six CGT → TGT, R → C and three CGT → CAT, R → H); in these tumours, the *p53* mRNA level was either 0.46 ± 0.22 AU when the wild-type allele was expressed or 0.87 ± 35 AU when not expressed. These results are identical to those obtained with respect to all mutated tumours expressing the wild-type allele (or not) (Table 2). Six of the missense mutations affected codon number 248 (four CGG → CAG, R → Q and two CGG → TGG, R → W), two of the missense mutations affected codon number 175 (CGC → CAC, R → H and CGC → GGC, R → G) and the codon number 245 (GGC → GGA, G → D and GGC → TGC, G → C). We also found two mutations at codon number 272 (GTG → ATG, V → M and GTG → TTG, V → L).

Eight mutations created major rearrangements of the mRNA reading frame (Table 4), four of them were insertions or deletions of one basepair. Deletions of cytosine 983 at codon 257 (tumour 147), adenine 1052 at codon 280 (tumour 164) and cytosine 1115 at codon 301 (tumour 9) gave three putative p53 proteins composed of 343 amino acids differing in their C-terminus. Insertion of a thymine after the guanine 989 at codon 259 (tumour 199) gave a putative shorter polypeptide composed of 262 amino acids. In four cases, more marked rearrangements were observed: a large rearrangement (tumour 121) in the N-terminus (from nucleotide 1 to 129), a non-homologous recombination of 43 basepairs after nucleotide 97 (tumour 44), a deletion of nine bases (amino acids 177 to 179, PHH) (tumour 194) and an insertion of five bases after nucleotide 1002 at codon 263 (tumour 155) giving a p53 protein composed of 343 amino acids. These eight rearrangements all concerned advanced Dukes' stage (seven were Dukes' stage C and one was Dukes' stage D).

### DISCUSSION

In this series of 109 primary colorectal carcinomas, we observed *p53* mRNA overexpression in 63% of the cases. This overexpression occurred without any correlation with the stage or site of the disease. The mean level of *p53* mRNA expression is five- to sixfold higher in these tumours than in adjacent normal mucosa. Some tumours showed high *p53* mRNA levels, while others showed a slight increase. In the latter case, *p53* mRNA may either be weakly expressed in all tumour cells or highly expressed in a few tumour cells owing to tumour heterogeneity. Indeed, it has been described that the tumour cell staining varied in extent and intensity when p53 protein is detected by IHC (*Zeng et al, 1994*). Our results clearly demonstrate that *p53* regulation may occur at a pretranslational step, involving either an increase in *p53* gene expression and/or stabilization of its mRNA. Our findings confirm earlier reports, which showed an elevated level of *p53* transcripts in 28 (Gope et al, 1991) and 25 (Lothe et al, 1992) cases, 70% and 66% of tested tumours overexpressed p53 respectively. In these studies, *p53* mRNA quantification was performed by Northern blot analysis.

When DNA sequencing was performed, 44 tumours (40%) exhibited *p53* mutation. Our data on the prevalence and spectra of *p53* mutations in colorectal cancer agree with those obtained from 960 cases compiled by Greenblatt et al (1994). However, the most striking difference between the two studies concerns the number of non-missense type mutations, which accounted for 18% of mutations in our study *vs* 8% in Greenblatt's study. Among the 36 substitutions of a single basepair, 28 of them occurred at CpG dinucleotides; the *p53* coding region contains 39 CpG dinucleotides, which are potential sites for the methylation of cytosine. Recently, Tornaletti et al (1995) found that the *p53* sequences along exons 5–8 were completely methylated at every CpG site, whatever the tissue. Methylation of CpG dinucleotides is thought to be the cause of the genetic changes occurring through spontaneous deamination of 5-methylcytosine (Rideout et al, 1990) and accounted for the majority of endogenous mutations in vertebrates (Sved et al, 1990). In our study, mutated and wild-type alleles were expressed simultaneously in 25 tumours, and the loss of wild-type allele expression was rarely observed in the proximal colon (25%) *vs* 50% and 43% in the distal colon and the rectum respectively. The rectum is a site in which a high incidence of 17p allelic loss has been observed. These differences were not statistically significant, and the hypothesis that genetic mechanisms leading to cancer differ in the proximal and distal colon needs to be verified (Delattre et al, 1989).

In 40 of the 44 mutated tumours, *p53* mRNA levels were significantly higher in tumours expressing the mutated allele alone than in tumours expressing both wild- and mutated-type alleles. It has been described that *p53* stimulates its own transcription (Deffie et al, 1993), and that some *p53* mutant forms presented wild-type transactivation activity (Levine et al, 1991). In addition, *p53* can induce transcription from an internal promoter located within the *mdm2* gene (Juven et al, 1993), whose product, MDM2 oncoprotein, has been identified as a negative regulator of the *p53* gene. Further, MDM2, by binding to *p53*, inhibits its ability to activate transcription and may, therefore, be part of a negative feedback loop serving to terminate signals involving the transient activation of wild-type *p53* (Oliner et al, 1992; 1993). So, when a *p53* gene mutation occurs, the absence of a negative feedback might be responsible for *p53* mRNA overexpression. The suppressor function of wild-type *p53* may also be compromised in cells containing a mutant allele of *p53*, since the formation of wild-type – mutant *p53* inactive complexes occurs (Milner et al, 1991). Thus, mutant forms abrogate the ability of wild-type *p53* to transactivate appropiate target genes in vitro and in vivo (Farmer et al, 1992; Kern et al, 1992), and the relative quantity of mutated to wild-type *p53* mRNA could determine the transformed phenotype, and the result could be partial or complete loss of wild-type function. *p53* mRNA overexpression can account for an elevated content of p53 protein in the absence of *p53* gene mutation. An increase in wild-type *p53* has been observed in colorectal adenomas and has been suggested to constitute an early event in the process of adenoma formation and carcinogenesis (Tominaga et al, 1993; Boccuzzi, 1995); our present data showing no correlation between *p53* mRNA and tumour stage agree with these results. Other results suggest that, in colorectal cancer, IHC detection of p53 protein does not always indicate the existence of an underlying *p53* gene mutation (Dix et al, 1994). This abnormal expression of wild-type p53 protein was also found in normal cells of a patient from a family with a history of cancer (Barnes et al, 1992) and, more recently, a new case of Li–Fraumeni was reported in which no mutation in the coding sequence of the *p53* gene was detected (Birch et al, 1994). In all cases, stabilization of the p53 protein depends on factors other than *p53* gene mutation, such as (1) binding to other molecules of cellular (*mdm2* gene product) or viral origin blocking *p53* in an inactive conformation; and/or (2) its

© Cancer Research Campaign 1997

sequestration in a cellular compartment in which it cannot exert its functions. Recent data have demonstrated wild-type p53 protein accumulation in the cytoplasm of astrocytomas (Lang et al, 1994), melanomas (Castresana et al, 1993) and testis cancer (Peng et al, 1993), and that nuclear exclusion of *p53* might also be one way of inactivating *p53* in breast cancer (Moll et al, 1992). Nuclear exclusion of wild-type *p53* is suggested in a study showing its cytoplasmic accumulation in colorectal cancers (Bosari et al, 1995) with a higher prevalence in advanced tumours (Sun et al, 1992). Another study has shown an increase in p53 protein according to the stage of carcinomas from the rectum (Starzynska et al, 1992). In our study, the level of *p53* mRNA did not correlate with either the Dukes' stage or the tumour site, in accordance with previous data showing no correlation in colorectal cancers between p53 overexpression detected by IHC and clinicopathological data (Yamaguchi et al, 1992, ; Bosari et al, 1994). This lack of correlation between p53 overexpression (either mRNA or protein) and tumour stage suggests that it is an early event in these tumours, perhaps beginning with adenoma formation (Tominaga et al, 1993).

*p53* mRNA overexpression with or without *p53* mutation suggests two distinct mechanisms of inactivation leading to the development of cancer, and that the *p53* status might have important implications for cancer therapy. The tumour-suppressing function of *p53* preserves genome integrity and the p53 protein is required for apoptosis in response to radiation-induced DNA damage, a mechanism serving to eliminate potentially oncogenic cells (Lee et al, 1993). The relation between *p53* mutations and the therapeutic response has been verified in vivo in athymic nude mice injected with embryonic transformed fibroblasts differing in their *p53* status. Tumours expressing the wild-type *p53* gene contained a high proportion of apoptotic cells and typically regressed after gamma radiation or doxorubicin treatment. In contrast, wild-type *p53*-deficient tumours continued to grow and contained few apoptotic cells (Lowe et al, 1994). These data show that much benefit could be gained from identifying cancers without *p53* mutations, which are likely to respond more favourably to drug therapy than those with mutated *p53*. Conversely, patients whose tumours harbour *p53* mutations might be spared from the toxicity associated with chemotherapy agents and would be good candidates for novel therapeutic approaches. Recently, Fujiwara et al (1994) reported the in vivo retroviral transduction of wild-type *p53* in human lung cancer cells in an orthotopic nude mouse model with endogenous mutated *p53*. They demonstrated that cancer cell growth can be eliminated or greatly reduced by this in vivo gene therapy beginning 3 days after tumour cell inoculation. So, 'cancer therapy meets *p53* status (Kinzler et al, 1994).

Analysis of the level of *p53* mRNA in colorectal cancer by quantitative RT–PCR provides a rapid and sensitive method for discriminating between tumours overexpressing *p53* mRNA with or without *p53* gene mutation. This should be useful for future anti-tumour research and for the design of therapeutic agents specific to the inactivation process. The observation period in our study was too short to clarify the relationship between *p53* mRNA overexpression and clinical prognosis of patients with a colorectal carcinoma; we are following these patients.

### ABBREVIATIONS

Abbreviations IHC, immunohistochemistry; RT, reverse transcription; PCR, polymerase chain reaction; dNPTs, deoxynucleotide triphosphates; DTT, dithiothreitol; AU, arbitrary units; SD, standard deviation.

### ACKNOWLEDGEMENTS

We are grateful to Biéna Bièvre-Marcé who edited the manuscript. We also thank Dr C Brahimi-Horn for critical reviewing.

### REFERENCES

Agoff SN, Hou J, Linzer DIH and Wu B (1993) Regulation of the human hsp 70 promoter by p53. *Science* **259**: 84–87

Auvinen A, Isola J, Visakorpi T, Koivula T, Virtanen S and Hakama M (1994) Overexpression of p53 and long-term survival in colon carcinoma. *Br J Cancer* **70**: 293–296

Baker SJ, Preisinger AC, Jessup JM, Paraskeva C, Markowitz S, Willson JKV, Hamilton S and Vogelstein B (1990) *p53* gene mutations occur in combination with 17p allelic deletions as late events in colorectal tumorigenesis. *Cancer Res* **50**: 7717–7722

Barnes DM, Hanby AM, Gillett CE, Mohammed S, Hodgson S, Bobrow LG, Leigh JM, Purkis T, MacGeoch C, Spurr NK, Bartek J, Vojtesek B, Picksley SM, and Lane DP (1992) Abnormal expression of wild-type p53 protein in normal cells of a cancer family patient. *Lancet* **340**: 259–263

Battifora H (1994) p53 immunohistochemistry: a word of caution. *Hum Pathol* **25**: 435–437

Birch JM, Heighway J, Teare MD, Kelsey AM, Hartley AL, Tricker KJ, Crowther D, Lane DP and Santibanez-Koref MF (1994) Linkage studies in a Li–Fraumeni family with increased expression of p53 protein but no germline mutation in p53. *Br J Cancer* **70**: 1176–1181

Boccuzzi A, Terzolo M, Leonardo E, Cappia S, Tappero G, Paccotti P and Angeli A (1995) High frequency of p53 expression in colorectal adenomatous polyps. Anticancer Res **15**: 1407–1410

Bosari S, Viale G, Bossi P, Maggioni M, Coggi G, Murray JJ and Lee AKC (1994) Cytoplasmic accumulation of p53 protein: an independent prognostic indicator in colorectal adenocarcinomas. *J Natl Cancer Instit* **86**: 681–687

Castresana JS, Rubio MP, Vazquez JJ, Idoate M, Sober AJ, Scizinger BR and Barnhill RL (1993) Lack of allelic deletion and point mutation as mechanisms of p53 inactivation in human malignant melanoma. *Int J Cancer* **55**: 562–565

Chin KV, Ueda K, Pastan I and Gottesman MM (1992) Modulation of activity of the promoter of the human *MDR-1* gene by ras and p53. *Science* **255**: 459–462

Clarke AR, Purdie CA, Harrison DJ, Morris RG, Bird CC, Hooper ML and Willye AH (1993) Thymocyte apoptosis induced by p53-dependent and independent pathways. *Nature* **362**: 849–852

Deffie A, Wu H, Reinke V and Lozano G (1993) The tumor suppressor p53 regulates its own transcription. *Mol Cell Biol* **13**: 3415–3423

Delattre O, Law DJ, Remvikos Y Sastre X, Feinberg AP, Olschwang S, Melot T, Salmon RJ, Validire P and Thomas G (1989) Multiple genetic alterations in distal and proximal colorectal cancer. *Lancet* **12**: 353–355

Dix B, Robbins P, Carrello S, House A and Iacopetta B (1994) Comparison of *p53* gene mutation and protein overexpression in colorectal carcinomas. *Br J Cancer* **70**: 585–590

Dulic V, Kaufmann WK, Wilson SJ, Tisty TD, Lees E, Harper JW, Elledge SJ and Reed SI (1994) p53-dependent inhibition of cyclin-dependent kinase activities in human fibroblasts during radiation-induced G1 arrest. *Cell* **76**: 1013–1023

El-Deiry WS, Kern SE, Pietanpol JA, Kinzler KW and Vogelstein B (1992) Definition of a consensus binding site for p53 *Nature Genet* **1**: 45–49

El-Deiry WS, Tokino T, Velculescu VE, Levy DB, Parsons R, Trent JM, Lin D, Mercer WE, Kinzler KW and Vogelstein B (1993) *WAF1*, a potential mediator of p53 tumor suppression. *Cell* **75**: 817–825

Faille A, De Cremoux P, Extra JM, Linares G, Espie M, Bourstyn E, De Rocquancourt A, Giachetti S, Marty M and Calvo F (1994) p53 mutations and overexpression in locally advanced breast cancers. *Br J Cancer* **69**: 1145–1150

Farmer G, Bargonetti J, Zhu H, Friedman P, Prywes R and Prives C (1992) Wild-type p53 activates transcription *in vitro*. *Nature* **358**: 83–86

Fearon ER and Vogelstein B (1990) A genetic model for colorectal tumorigenesis. *Cell* **61**: 759–767

Fritsch EF, Sambrook J and Maniatis T (1989) *Molecular Cloning. A Laboratory Manual*, 2nd edn. pp 916–919 Cold Spring Harbor Laboratory Press: Cold Spring Harbor, NY

Fujiwara T, Cai DW, Georges RN, Mukhopadhyay T, Grimm EA and Roth GJ (1994) Therapeutic effect of a retroviral wild-type p53 expression vector in an orthotopic lung cancer model. *J Natl Cancer Inst* **86**: 1458–1459

Funk WD, Pak DT, Karas RH, Wright WE and Shay JW (1992) A transcriptionally active DNA-binding site for human p53 protein complexes. *Mol Cell Biol* **12**: 2866–2871

© Cancer Research Campaign 1997

Ginsberg D, Mechta F, Yaniv M and Oren M (1991) Wild-type p53 can down-modulate the activity of various promoters. *Proc Natl Acad Sci USA* **88**: 9979–9983

Gope M L, Chun M and Gope R (1991) Comparative study of the expression of *Rb* and *p53* genes in human colorectal cancers, colon carcinoma cell lines and synchronized human fibroblasts. *Mol Cell Biol* **107**: 55–63

Greenblatt MS, Bennett WP, Hollstein M and Harris CC (1994) Mutations in the p53 tumor supressor gene: clues to etiology and molecular pathogenesis. *Cancer Res* **54**: 4855–4878

Hamelin R, Laurent-Puig P, Olschwang S, Jego N, Asselain B, Remvikos Y, Girodet J, Salmon RJ and Thomas G (1994) Association of p53 mutations with short survival in colorectal cancer. *Gastroenterology* **106**: 42–48

Harper JW, Adami GR, Wei N, Keyomarsi K and Elledge SJ (1993) The p21 Cdk-interacting protein Cip1 is a potent inhibitor of G1 cyclin-dependent kinases. *Cell* **75**: 805–816

Hollstein M, Sidransky D, Vogelstein B and Harris CC (1991) *p53* mutations in human cancers. *Science* **253**: 49–53

Horio Y, Takahashi T, Kuroishi T, Hibi K, Suyama M, Niimi T, Shimokata K, Yamakawa K, Nakamura Y, Ueda R and Takahashi T (1993) Prognostic significance of *p53* mutations and 3p deletions in primary resected non-small cell lung cancer. *Cancer Res* **53**: 1–4

Juven T, Barak Y, Zauberman A, George DL and Oren M (1993) Wild type p53 can mediate sequence-specific transactivation of an internal promoter within the *mdm2* gene. *Oncogene* **8**: 3411–3416

Kastan MB, Onyekwere O, Sidransky D, Vogelstein B and Craig RW (1991) Participation of p53 protein in the cellular response to DNA damage. *Cancer Res* **51**: 6304–6311

Kastan MB, Zhan Q, El-Diery WS, Carrier F Jacks T, Walsh WV, Plunkett BS, Vogelstein B and Fornace AJ Jr (1992) A mammalian cell cycle checkpoint pathway utilizing p53 and *GADD45* is defective in ataxia-telangiectasia. *Cell* **71**: 587–597

Kennedy SM, MacGeogh C, Jaffe R and Spurr NK (1994) Overexpression of the oncoprotein p53 in primary hepatic tumors of childhood does not correlate with gene mutations. *Hum Pathol* **25**: 438–442

Kern SE, Fearon ER, Tersmette KWF, Enterline JP, Leffert M, Nakamura Y, White R, Vogelstein B and Hamilton S R (1989) Allelic loss in colorectal cancer. *JAMA* **261**: 3099–3103

Kern SE, Kinzler KW, Bruskin A, Jarosz D, Friedman P, Prives C and Vogelstein B (1991) Identification of p53 as a sequence-specific DNA-binding protein. *Science* **252**: 1708–1711

Kern SE, Pietenpol JA, Thiagalingam S, Seymour A, Kinzler W and Vogelstein B (1992) Oncogenic forms of p53 inhibit p53-regulated gene expression. *Science* **256**: 827–830

Kinzler KW and Vogelstein B (1994) Clinical implications of basic research: cancer therapy meets p53. *N Engl J Med* **131**: 49–50

Kupryjanczyk I, Thor AD, Beauchamp R, Merritt V, Edgerton SM, Bell DA and Yandell DW (1993) *p53* gene mutations and protein accumulation in human ovarian cancer. *Proc Natl Acad Sci USA* **90**: 4961–4965

Lane DP (1992) p53, guardian of the genome. *Nature* **358**: 15–16

Lang FF, Miller DC, Pisharody S Koslow M and Newcomb E W (1994) High frequency of p53 protein accumulation without *p53* gene mutation in human juvenile pilocytic, low grade and anaplastic astrocytomas. *Oncogene* **9**: 949–954

Lee JM and Bemstein A (1993) p53 mutations increase resistance to ionizing radiation. *Proc Natl Acad Sci USA* **90**: 5742–5746

Levine AJ, Momand J and Finlay CA (1991) The p53 tumour suppressor gene. *Nature* **351**: 453–455

Liu X, Miller CW, Koeffler PH and Berk AJ (1993) The p53 activation domain binds the TATA box-binding polypeptide in Holo-TFIID, and a neighboring p53 domain inhibits transcription. *Mol Cell Biol* **13**: 3291–3300

Lothe RA, Fossli T, Danielsen HE, Stenwig AE, Nesland JM, Gallie B and Borressen AL (1992) Molecular genetic studies of tumor suppressor gene regions on chromosomes 13 and 17 in colorectal tumors. *J Natl Cancer Inst* **84**: 1100–1108

Lowe SW, Schmitt EM, Smith SW, Osborne BA and Jacks T (1993) p53 is required for radiation-induced apoptosis in mouse thymocytes. *Nature* **362**: 847–849

Lowe SW, Bodis S, McClatchey A, Remington L, Ruley HE, Fisher DE, Housman DE and Jacks T (1994) p53 status and the efficacy of cancer therapy *in vivo*. *Science* **266**: 807–810

Marklin D, Li FP, Strong LC, Fraumeni JF, Jr, Nelson CE, Kim DH, Kassel J, Cryka MA, Bischoff FZ, Tainsky MA and Friend SH (1990) Germ-line p53 mutations in a familial syndrome of breast cancer, sarcoma and other neoplasms. *Science* **250**: 1233–1238

Milner J, Medcalf AE and Cook CA (1991) Tumor suppressor p53: analysis of wild-type and mutant p53 complexes. *Mol Cell Biol* **11**: 12–19

Moberg KH, Tyndall WA and Hall DJ (1992) Wild-type murine p53 represses transcription from the murine c-*myc* promoter in a human glial cell line. *J Cell Biol* **49**: 208–215

Moll UM, Riou G and Levine AJ (1992) Two distinct mechanisms alter p53 in breast cancer: mutation and nuclear exclusion. *Proc Natl Acad Sci USA* **89**: 7262–7266

Oliner JD, Kinzler KW, Meltzer PS, George DL and Vogelstein B (1992) Amplification of a gene encoding a p53-associated protein in human sarcomas. *Nature* **358**: 80–83

Oliner JD, Pietenpol JA, Thiagaligam SS, Gyuris J, Kinzler KW and Vogelstein B (1993) Oncoprotein MDM2 conceals the activation domain of tumor supressor p53. *Nature* **362**: 857–860

Papathanasiou MA, Kerr NC, Robbins JH, McBride OW, Alamo I Jr, Barrett SF, Hickson ID and Fornace AJ Jr (1991) Induction by ionizing radiation of the *gadd-45* gene in cultured human cells: lack of mediation by protein kinase C. *Mol Cell Biol* **11**: 1009–1016

Peng HQ, Hogg D, Malkin D, Bailey D, Gallu BL, Bultal M, Jerrett M, Buchanan J and Cross PE (1993) Mutation of the *p53* gene did not occur in testis cancer. *Cancer Res* **53**: 3574–3578

Pignatelli M, Stamp GW, Kafiri G, Lane D and Bodmer WF (1992) Over-expression of p53 nuclear oncoprotein in colorectal adenomas. *Int J Cancer* **50**: 683–688

Rideout WM III, Coetzee GA, Olumi AF and Jones PA (1990) 5-Methylcytosine as an endogenous mutagen in the human LDL receptor and p53 genes. *Science* **249**: 1288–1290

Santhanam U, Ray A and Sehgal PB (1991) Repression of the interleukin 6 gene promoter by p53 and the retinoblastoma susceptibility gene product. *Proc Natl Acad Sci USA* **88**: 7605–7609

Shiio Y, Yamamoto T and Yamaguchi N (1992) Negative regulation of Rb expression by the p53 gene product. *Proc Natl Acad Sci USA* **89**: 5206–5210

Shin DM, Kim J, Ro JY, Hittelman J, Roth JA, Hong WK and Hittelman WN (1994) Activation of *p53* gene expression in premalignant lesions during head and neck tumorigenesis. *Cancer Res* **54**: 321–326

Srivastava S, Zou Z, Pirollo K, Blattner W and Chang EH (1990) Germ-line transmission of a mutated *p53* gene in a cancer-prone family with Li–Fraumeni syndrome. *Nature* **348**: 747–749

Starzynska T, Bromley M, Ghosh A and Stern PL (1992) Prognostic significance of p53 overexpression in gastric and colorectal carcinoma. *Br J Cancer* **66**: 558–562

Subler MA, Martin DW and Deb S (1992) Inhibition of viral and cellular promoters by human wild type p53. *J Virol* **66**: 4757–4762

Sun XF, Carstensen JM, Zhang H, Stal O, Wingren S, Hatschek T and Nordenskjöld B (1992) Prognostic significance of cytoplasmic p53 oncoprotein in colorectal adenocarcinoma. *Lancet* **340**: 1369–1373

Sved J and Bird A (1990) The expected equilibrium of the CpG dinucleotide in vertebrate genomes under a mutation model. *Proc Natl Acad Sci USA* **87**: 4692–4696

Thorlacius S, Börresen AL and Eyfjörd JE (1993) Somatic *p53* mutations in human breast carcinomas in an icelandic population: a prognostic factor. *Cancer Res* **53**: 1637–1641

Thut CJ, Chen JL, Klemm R and Tjian R (1995) p53 transcriptional activation mediated by coactivators $TAF_{II}40$ and $TAF_{II}60$. *Science* **267**: 100–103

Tominaga O, Hamelin R, Trouvat V, Salmon RJ, Lesec G, Thomas G and Remvikos Y (1993) Frequently elevated content of immunochemically defined wild-type p53 protein in colorectal adenomas. Oncogene **8**: 2653–2658

Tornaletti S and Pfeifer GP (1995) Complete and tissue-independent methylation of CpG sites in the *p53* gene: implication for mutations in human cancers. *Oncogene* **8**: 1493–1499

Vogelstein B (1990) A deadly inheritance. *Nature* **348**: 681–682

Waga S, Hannon GJ, Beach D and Stillman B (1994) The p21 inhibitor of cyclin-dependent kinases controls DNA replication by interaction with PCNA. *Nature* **369**: 574–578

Wagata T, Shibagaki I, Imamura M, Shimada Y, Toguchida J, Yandell DW, Ikenaga M, Tobe T and Ishizaki K (1993) Loss of 17p, mutation of the *p53* gene and overexpression of p53 protein in esophageal squamous cell carcinomas. *Cancer Res* **53**: 846–850

Weintraub H, Hauschka S and Tapscott S J (1991) The MCK enhancer contains a p53 responsive element. *Proc Natl Acad Sci USA* **88**: 4570–4571

Wolkmann M, Holmann WJ, Müller M, Räth U, Otto G, Zentgraf H and Galle PR (1994) p53 overexpression is frequent in European hepatocellular carcinoma and largely independent of the codon 249 hot spot mutation. *Oncogene* **9**: 195–204

© Cancer Research Campaign 1997

Yamaguchi A, Kurosaka Y, Fushida S, Kano M, Yonemura Y, Miwa K and Miyazaki I (1992) Expression of p53 protein in colorectal cancer and its relationship to short-term prognosis. *Cancer* **70**: 2778–2784

Zambetti GP and Levine AJ (1993) A comparison of the biological activities of wild-type and mutant p53. *FASEB J* **7**: 855–863

Zeng ZS, Sarkis AS, Zhang ZF, Klimstra DS, Charutonowicz E, Guillem JG, Cordon-Cardo C and Cohen AF (1994) p53 nuclear overexpression: an independent predictor of survival in lymphnode-positive colorectal cancer patients. *J Clin Oncol* **12**: 2043–2050

*© Cancer Research Campaign 1997*